# Operative Procedure in Traumatic Epilepsy

**Published:** 1889-01

**Authors:** J. T. Wilson

**Affiliations:** Sherman, Texas


### OPERATIVE PROCEDURE IN TRAUMATIC EPILEPSY.*

BY J. T. WILSON, M. D., SHERMAN, TEXAS.

Reflex epilepsy from traumatic injuries of the brain is not a rare trouble, and nearly every surgeon meets with one or more cases during his professional life.

I believe it to be proportionately the most curable of epilepsies. It is a very important affection and requires more study from the general practitioner than is usually given it. The great mistake is too frequently made by either not operating at all or waiting too long. In truth the tendency in these cases is too frequently to neglect them, or prescribe only a palliative treatment. In this day of rapid surgical advance, when the hitherto sacred precinct of the cranium can be invaded with so much less hesitation than in former years, and with such comparative safety since the introduction of antiseptics, and with the brilliant record of brain surgery before us, where tumors and otherpathological lesions are diagnosticated and removed with such splendid results as to astonish the world, no case of epilepsy caused by a depressed fracture should be permitted to progress without giving the unfortunate and wretched victim an opportunity to receive the benefits an operation might bring. Trephining does not aggravate the

*Read before the Southern Surgical and Gynecological Association.

convulsions, and even if no benefit should result it does not leave the patient in a worse condition, when he has been properly prepared for it and the operation is carefully done under strict modern rules.

Though hemiplegia exist and its liability to remain as taught by the English surgeons should not deter us from operating and curing the fits if possible. In my limited experience with this disease the convulsions in a majority of cases are extremely severe, sometimes quite frequent and usually to a great extent one sided, that is, the muscles of one side are nearly all involved in the spasm, though not in the same degree; the muscles of the face and upper extremity of that side are in most cases more affected than those of the trunk and lower extremity. Antispasmodics generally have less control over them than over the attacks from other causes, and their controlling influence does not last so long. In a majority of the cases that I have seen the general health was fairly good; they had morbid appetites and ate ravenously large quantities of food, and in all the cases coming under my observation, to the best of my recollection, the frontal, temporal and anterior region of the parietal bones sustained the injuries.

General treatment, attention to the general health with tonics, antispasmodics, and more especially the bromides, would always lessen the severity and to some extent the frequency of the attacks for a while; but the bromides largely given for any considerable length of time seemed to have a deleterious effect upon the health of this class of cases, and also seemed to have a more unfavorable influence upon the mental state, inducing early dementia. Persons predisposed to epilepsy or very nervous and excitable, who are very susceptible to nervous shocks, are more liable to epilepsy from fracture or other injuries of the skull and scalp than those who are differently constituted.

In one patient a slight depression will produce it, in another a deep one will not, though both occur in the same locality, and in these nervous cases especially is an operation likely to do good, but in all it is of paramount importance to do the operation thoroughly.

In this connection I will report two cases illustrative of the object I have in view, that taught me an important lesson in regard to operating upon the skull and brain for epilepsy caused by an injury, and the lessons there learned served me a useful purpose in other cases.

*Case* I.—J. H. O., American, male, age 40, married, occupation farmer. No reliable family history could be obtained nor of himself, except that he had always been very nervous and excitable. General health fair, though not robust, of very nervous temperament. Was affected with chronic melancholia. Attempted suicide by cutting through his skull with the sharp corner of an axe, the wound being through the coronal suture, involving the frontal and parietal bones and about half an inch above the temporal bone of right side. Not succeeding by this means, he made another attempt a few days later by driving a No. 6 nail through the opening in the skull made with the axe. This nail was driven for more than an inch into the cerebral tissue. A physician saw him soon after and extracted it, dressed the wound by the ordinary, old-fashioned method; some suppuration supervened and the wound closed by granulation, paralysis of left side gradually came on; in six weeks was almost complete. A few weeks after the closure of the wound he was attacked with epileptic convulsions and they recurred at intervals of one to three weeks for several months, and as time passed they grew more frequent and severe. Bromides in full doses seemed for a while to lessen the severity but not the frequency of the attacks, and finally seemed to lose all effect over them, and after the use of them for several months his digestion began to suffer, and symptoms of beginning dementia were strongly marked, but strange to say the paralysis slowly improved sufficiently to enable him to walk about with the assistance of a cane. At the end of nine months from the reception of the injury the bromides were discontinued, he was put upon a tonic regimen for about two weeks, when he was anæsthetized, the scalp shaved and a crucial incision made over the old cicatrix; as the scalp was dissected up and drawn back, there was found a small oblong depression of the skull, and in the centre a small opening leading down to the dura mater, the bone having sloughed away. The trephine was used and two buttons removed embracing the entire length of the depression. A thick cicatrix of the dura mater was observed, but it looked healthy and it was thought the epilepsy was caused by the depressed bone, it was therefore not interfered with, and after all the depressed bone was removed the wound was closed by the ordinary method, it healed readily under cold water dressings. He improved rapidly and for three months had no convulsion, his general health improved, paralysis improved so much that he could walk comparatively well with the assistance of a cane and go wherever he desired, his mental condition also mended considerably, was more cheerful than he had been for months, and it was thought that his recovery would in a short time be complete. After the lapse of three months he began to get somewhat nervous and morose, appetite decreased and paralysis increased, his melancholia began to be markedly observed, he was restless, though not inclined to walk much, was troubled with insomnia and complained of pain in his head at times that was quite severe. A marked change in his general condition was easily observed. This change came on rapidly and one morning after waking from sleep he was attacked with a very severe convulsion which lasted nearly two hours and from which he never rallied but sank and died in three or four hours. The autopsy revealed a very much contracted calibre of the trephined wound, it having filled up with new tissue. The old cicatrix of the membranes was hard and firm, bulging a little and adherent to the skull around the wound. When it was incised an abscess of the brain was revealed in which was found a small spicula of bone about half an inch in length. The brain tissue was softened and slightly discolored for perhaps a sixth of an inch around, extending into the cerebral tissue from the abscess cavity.

If when this patient was trephined the cicatrix in the membrane had been cut away, the part explored, the piece of bone removed and the diseased brain scraped away, it is highly probable that his epilepsy and perhaps his paralysis might have been cured and his life prolonged.

*Case* II.—T. M., American, male, age 24, farmer. Fell from a tree a distance of over twenty feet, striking his head upon a hard substance and sustaining a fracture of the skull involving the frontal bone of the left side near its junction with the parietal. According to the history obtained he was said to have remained insensible for several hours and confined to his bed for nearly three weeks. He gradually recovered and in the fifth week after the accident and after a day's labor of unusual severity, was attacked with epilepsy followed by another seizure the day after. Another occurred after an interval of two weeks, then continued at irregular intervals for fifteen months, the attacks growing more frequent and severe, any unusual mental and physical exertion bringing them on. Very little benefit obtained from internal treatment, his digestion was much disturbed by large doses of bromides, he had a morbid appetite, his intellect was dulled, and his case presented a gloomy prospect. After about ten days' preparation he was trephined, two buttons of bone removed, a small piece of the internal table lying loose was taken away, the wound closed up and healed without trouble, he was confined to his bed for ten days and in fifteen days after the operation left his room. He seemed to be doing well until twenty-one days after the operation when he had a mild epileptic attack, and these attacks continued with variable frequency and severity for four months, when it was determined to again resort to operative procedure which was accordingly done. A U shaped incision was made, flap turned back and two buttons of bone removed from the sides of the old wound. A tense cicatrix of the dura mater presented and was carefully removed entire. The brain beneath presented no perceptible pathological lesion, the wound was washed with warm carbolized water, the scalp dissected up for nearly an inch around the wound, edges brought together with silk suture and closed up. The patient recovered without an untoward symptom, had five or six mild spasms in the following three months when they ceased altogether, and after five years—when heard from a few months ago—has had no further trouble but remains well and able to cultivate his farm.

These operations were not done under strict antiseptic rules, as the practice had not come into such general use as at the present day; but in the second case warm carbolized water was used. This experience taught me that in all cases of trephining when the bone is removed the part of brain and membranes exposed should be carefully examined, and if a cicatrix exists it should be removed, as also any foreign substance or pathological lesion of the brain should be cut or scraped away, under thorough antiseptic precautions. If hemorrhage occurs, and continues after washing with hot water, and if bleeding vessel or vessels cannot be ligated, the cavity can be very carefully and lightly packed with antiseptic wool, care being taken that the packing be not too tight, as the pressure thus made might result in mischief, and as soon as the hemorrhage has been controlled it should be removed.

It is not the pressure of the bone in every instance that causes the reflex disturbance, for the brain will sometimes become accustomed to this pressure, if not too great, and its functions go on as before after recovering from the shock of the accident, but if the membranes have been lacerated at the time, or sustained some other injury followed by a circumscribed meningitis, a cicatrix will form, possibly adhesions and some contraction take place, the cortical substance will be irritated and from this cause convulsions are liable to ensue, or circumscribed inflammation may extend to the brain and abscess result, or a spicula of bone may be driven into the cerebrum and its presence excite the disease, therefore these sources of irritation should always be looked for and removed. Mr. Horsley thinks that after a button is removed, if it is noticed that the dura mater bulges through the opening, it is an indication of the existence of pathological intercranial tension.

I believe in all cases of epilepsy, caused by depression from an accident, a contracted cicatrix of the scalp or a sensitive spot in the head (as in a case that came under my observation some years ago, which by pressure upon a sensitive spot would produce convulsions), trephining is not only justifiable but demanded and should be done without hesitation. If the bone is thickened Professor Briggs'* teaching is correct, remove it; trephine a second and a

*International Encyclopœdia of Surgery.

third time, if need be, taking away all the diseased bone, or even though it be not diseased, if there is much unnatural thickness remove it, and if any pathological condition of the membranes of the cerebrum be found it should without hesitation be carefully cut away, leaving only healthy tissue, especially as regards the cortical substance and no tense, thick cicatrix of the *membranes should be allowed* to remain. Even thick, sensitive cicatrices of the scalp, in my opinion, should be removed, if there be the slightest suspicion that the epilepsy is caused by the traumatism.

After these operations the healing is materially assisted, and subsequent danger lessened, by dissecting up the scalp for a few lines around the wound in the skull before it is brought together, in order not to have too much tension by cicatrictial contraction. The wound should be carefully dressed by the antiseptic method and watched, every source of supposed irritation removed, and if possible, made to heal by first intention to prevent an extensive scar. The general health should be looked after, secretive functions kept active and nervous system tranquilized, the patient kept quiet and cheerful, away from all sources of excitement and from everything that would in any way affect the general health, for several weeks after recovery seems complete. In my judgment much of the success in many cases depends upon the proper attention to careful and judicious after treatment.

In long standing cases improvement in the convulsions does not always begin at once; sometimes several months elapse before the improvement is marked and the habit gradually broken up, for there is a good deal in the habit the brain sometimes gets into with regard to these convulsions. Many neurologists now think that "idiopathic epilepsy has its origin in the cortex of the cerebrum and from a surgical standpoint it is therefore not difficult to understand that any chronic endo-cranial lesion which affects this part of the brain can produce epilepsy." Dr. Senn,* of Milwaukee, in his investigations recognizes as causes of reflex epilepsy affections or wounds of the peripheral nerves, which indirectly affect the cortex of the brain as well as lesions of the cortex itself.

*Annual Universal Medical Sciences Vol. II, 1888.

It sometimes occurs that in trephining the skull for injuries or disease the surgeon is not careful enough in his explorations of the wound, and fails to remove splinters of bone or other foreign substance, which is liable to cause an inflammatory condition and may produce epilepsy among other troubles.

Is it too much to hope that surgery may yet come to the aid of many epileptics who are otherwise considered incurable, and doomed to a life of untold misery, whether their trouble be of traumatic origin or not?

